# ECMO-assisted resection of huge thoracic mass 

**DOI:** 10.15171/jcvtr.2018.28

**Published:** 2018-05-21

**Authors:** Alireza Jahangirifard, Zargham Hossein Ahmadi, Abolghasem Daneshvar Kakhaki, Behrooz Farzanegan, Kambiz Sheikhy

**Affiliations:** ^1^Lung Transplantation Research Center, National Research Institute of Tuberculosis and Lung Diseases (NRITLD), Shahid Beheshti University of Medical Sciences, Tehran, Iran; ^2^Tracheal Diseases Research Center, National Research Institute of Tuberculosis and Lung Diseases (NRITLD), Shahid Beheshti University of Medical Sciences, Tehran, Iran

**Keywords:** ECMO, Huge Thoracic Mass, Hemodynamic, Oxygenation

## Abstract

Some advanced thoracic malignancy cannot be resected safely by using of conventional ventilation, so some sort of cardiopulmonary support is needed for hemodynamic and ventilation management of the patient. Using extracorporeal membrane oxygenation (ECMO) in comparing with cardiopulmonary bypass has some advantages. Three patients with huge thoracic tumors with different ages experienced major surgery in our center by using ECMO in order to face major complications mainly due to the size of mass to achieve better hemostatic stabilities, lower bleeding, and injuries to main airways and secure oxygenation. This is the first case series in Iran, as our best knowledge that explains cases of huge chest mass which were operated perfectly by using ECMO and short ICU stay and interestingly no major complications.

## Introduction

More than 40 years have been elapsed from the first ideas and versions of extracorporeal membrane oxygenation (ECMO) which was designed as a cardiopulmonary support in adults with multiple indications from respiratory failure management to cardiopulmonary transplantation. Severe respiratory failure with hypoxemia or hypercapnia which responses to mechanical ventilation insufficiently is the main indication for ECMO appliance. One of the best devices ever introduced for the medical approach in these patients is ECMO which affects O2 and CO2 exchange simultaneously.^1,2^ ECMO has grown more to be applied since 2009 for respiratory failure cases although it is a perfect alternative for cardiopulmonary bypass during lung transplantation and other cardiothoracic surgery for managing hemodynamic stability and perfect oxygenation.^3^ Mechanical ventilation could also be avoided at the time of complex thoracic surgeries that makes ECMO more popular, especially in operations like huge mass excision, major airways surgery and lung transplantation. Excision of huge thoracic mass is mainly difficult by using conventional mechanical ventilation and needs some type of cardiopulmonary support. By the current report, we are introducing three cases of thoracic masses that were actually difficult to manage without ECMO. We used ECMO via femoral artery and vein at the beginning of surgery. After successful resection of tumor, flow of ECMO was reduced gradually till it reached 30% of cardiac output and in the presence of hemodynamic stability and ideal oxygen saturation, ECMO discontinued but cannulas remained for about 30 minutes and finally after continuing stability of patient were removed.

## Case Report

### Case 1

**Figure 1:**
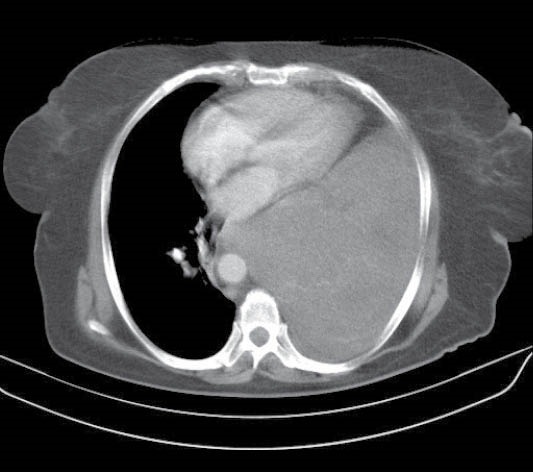
Left huge thoracic mass with deviation of mediastinal viscera

A 59-year-old woman was referred with the history of exertional dyspnea. She also had left-sided chest pain and BIPAP ventilation device dependence. Primary studies found a huge mass in left hemi thorax (Figure 1). The CT-guided biopsy had been done for the patient and solitary fibrous tumor was the final pathologic diagnosis. Open surgical excision was planned. Except for the breathing rate of 20/minute, vital signs were normal. The pronounced disorder was seen in PFT and FEV1 that was only 20% predicted value. The patient underwent general anesthesia before connecting to ECMO machine. ECMO was used via femoral artery and vein through Seldinger technique. After positioning of the patient to lateral decubitus marked hemodynamic and oxygenation derangement occurred so operation continued by the assistance of ECMO. The huge mass was excised via posterolateral thoracotomy. Complete excision was uneventful and during operation, the patient was completely stable. ECMO was continued for about 3 hours postoperatively to assure hemodynamic and blood gas stability before weaning. The mass sized 18×16×10 cm with 1670 g weight. There was no major complication and O2 saturation was 94% immediately after the operation. She extubated 12 hours after ICU transferring. There was no major post-operative complication and liver function tests (LFT) were in normal range and except in mild anemia that managed conservatively there was no other abnormality in laboratory tests. At 10-month follow up there was no problem and the patient has no dyspnea and lungs were completely expanded and there was no signs and symptoms of tumor recurrence.

### Case 2

**Figure 2:**
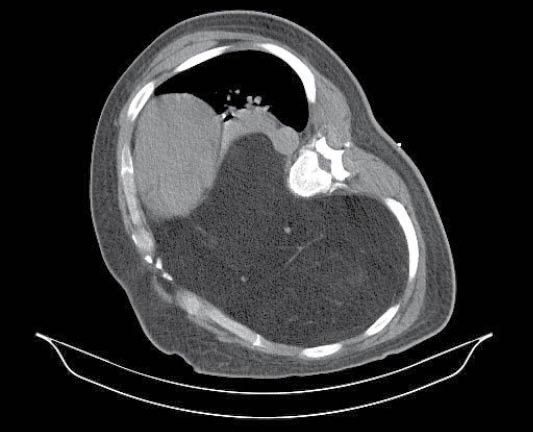
Huge right thoracic mass. Patient could not tolerate supine position at the time of CT scanning.

A 45-year-old man with the 2 years history of exertional dyspnea, dry cough and a huge mass in right hemi thorax was referred to our center. The mentioned mass had a fatty density at CT scan while shifted the heart to the left (Figure 2). CT guided biopsy revealed the diagnosis of lipoma. FEV1was 0.65liter and respiratory rate were 20/minute but other vital signs were within normal range. Due to huge mediastinal soft tissue mass (lipoma) and pleural effusion along with the mentioned symptoms, surgical resection was planned for the patient. According to low oxygen saturation and dimension of mass, ECMO was used at the beginning of the operation. Cannulation was done from right femoral artery and vein by a cardiac surgeon after anesthesia induction to use ECMO. At the time of operation, a very large ovoid encapsulated tumor with fat consistency was excised through a posterolateral thoracotomy. He had an O2 saturation of 96% and his condition was stable postoperatively so using of ECMO discontinued at the end of surgery. After six hours, right sided re-expansion pulmonary edema developed which was managed by intubation and mechanical ventilation. Early post-operative anemia managed by packed cell transfusion although there was no major bleeding at this time. All other laboratory tests were normal. The patient discharged two weeks after surgery after successful weaning and discontinuing mechanical ventilation. The final pathologic report confirmed the diagnosis of low-grade liposarcoma. According to oncology consult the patient did not need adjuvant therapy. Nearly after two years of surgery the patient is completely symptomless and there is no tumor recurrence.

### Case 3

**Figure 3:**
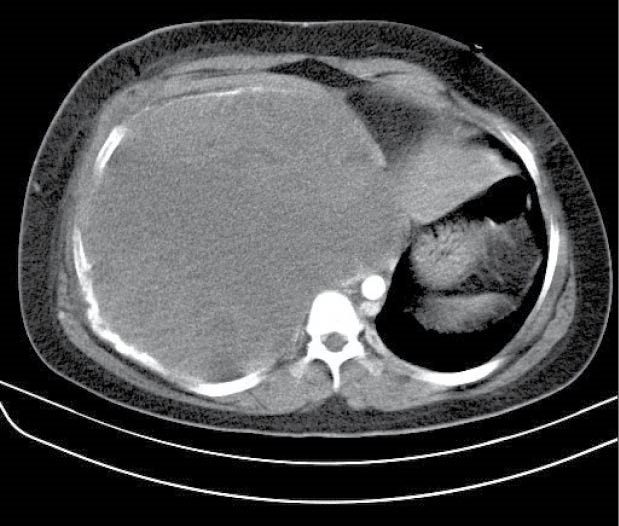
Obliteration of right hemithorax by very large mass.

A 13-year-old boy with a one-month history of chronic cough in addition to dyspnea referred by oncology service. Vital signs and O2 saturation were rather normal except the respiratory rate of 26/minute. CT scan was done that showed a huge mass with compression effects on the heart (Figure 3). CT guided biopsy was undiagnostic so we performed an incisional biopsy that was reported aneurysmal bone cyst. At echocardiography, there was observable compression of the mass on right atrium and inferior vena cava. Peripheral ECMO was applied through cannulation of left femoral artery and vein under general anesthesia. After ECMO installation a wide posterolateral thoracotomy was done. Diaphragmatic and pulmonary adhesion with pulmonary collapse were seen. Large hemorrhagic tumor was excised completely with adjacent seventh and eighth ribs as the origin of the tumor. The patient was stable after the surgery with 100% O2 saturation without the need of using ECMO in the postoperative period. According to hemorrhagic nature of tumor, bleeding was modest at the time of operation but after complete resection of the tumor, there was no major bleeding but due to low hemoglobin level (Hg=6 mg/dL) packed cell transfused. He was extubated in the operating room after ECMO removal and finally was discharged on the sixth postoperative day. The final pathologic diagnosis was compatible with telangiectatic osteosarcoma and at the present time adjuvant chemotherapy has been administered for him by oncologist.

## Discussion

Resection of advanced thoracic malignancy is always challenging for surgeons that usually originate from the complexity of airway or vascular resection. Although hemodynamic stability is another major concern due to compressive effect of these masses or prolonged surgical manipulation. This case series explains advantages of ECMO in surgeries through which huge thoracic masses were excised when compression effects and the risk of local injuries to airways or great vessels are the main concern. In our patients there was marked compressive effect of tumor on heart and large thoracic vessels so hemodynamic instability especially at the time of lateral decubitus positioning was the main concern. Also according to this large dimensions one hemithorax was totally occluded and tumor was protruded to opposite side so patients had some degree of respiratory problems and with considering these problems using conventional ventilation was inappropriate for resection. Resection of advanced thoracic malignancies or large masses had been always a challenge when conventional ventilation techniques were used which was almost resolved by cardiopulmonary bypass (CPB) that provides oxygenation and hemodynamic stability at the same time. However, despite its perfect effectiveness, more needed blood products following full heparinization of the patient, the risk of tumor cell spilling into the suction system and potential immunosuppressive effects of the device downgrade the value of CPB, especially in cases with huge mediastinal mass resection.^4^ No need to full heparinization and suction as well as better biocompatibility, ECMO has been more attractive for surgeons in these situations.^5^ ECMO system is heparin-coated and intraoperative anticoagulation is done by only 70 IU/kg body weight heparin for the patient. Furthermore, ECMO has a closed circulatory system with no contact with a suction system in surrounding tissues which contain tumor and normal cell debris. A clean site of operation is always important to keep surgeon very accurate to have a precise dissection, especially in limited spaces like the chest that is truly provided by ECMO. Although Hoechter et al explained that ECMO had no advantages compared with CPB in case of lung transplantation and intraoperative blood transfer rate through their meta-analysis, they disclose that ECMO could reduce ICU stay and was superior in terms of 3-month and one-year survive concluding that ECMO has benefits in its intraoperative use, especially in short-term outcome.^6^ This is while Sharma et al. have more positive concepts of using ECMO in lung transplantation.^7^ In terms of morbidity and mortality, Lang et al presented their experience with ECMO on nine patients among them one developed the only ECMO-related problem in their report, lymphatic fistula at the groin, which needed surgical revision. They also reported one case of perioperative death.

This is the first report in Iran, as far as our best knowledge, that explain cases of huge thoracic masses which were operated perfectly using ECMO with shorter ICU stay for patients and interestingly no major complications.

## Ethical approval

Informed consent was obtained from the participants for publication of this case report.

## Competing interests

All authors declare no competing financial interests exist.
